# Fungal keratitis caused by *Scedosporium apiospermum*: a case report

**DOI:** 10.1186/s13256-022-03566-6

**Published:** 2022-09-07

**Authors:** Çisil Erkan Pota, Yusuf Ayaz, Mustafa Ünal, Özlem Koyuncu Özyurt

**Affiliations:** 1 Department of Ophthalmology, Faculty of Medicine, Akdeniz University, Pınarbaşı Mah. Akdeniz Üniversitesi Tıp Fakültesi Hastanesi, Antalya, Turkey; 2 Department of Microbiology, Faculty of Medicine, Akdeniz University, Antalya, Turkey

**Keywords:** Anti-infective agents, Cornea, Diseases of the ocular surface, Fungal keratitis, Keratitis

## Abstract

**Background:**

We present a case of fungal keratitis caused by *Scedosporium apiospermum*, which is a rare agent.

Case description

A 34-year-old Caucasian male patient was admitted to our clinic with complaints of pain and blurred vision in the left eye. The patient had a history of wearing contact lenses for 3 years. According to the Snellen chart, the patient’s visual acuity was 20/20 and counting fingers at 30 cm, for right and left eyes, respectively. A 3 × 3 mm corneal abscess at the center of the cornea with hypopyon in the patient’s left eye was observed. After the patient was hospitalized, fortified gentamicin and fortified cefazolin drops were started 24 times per day. Intravenous fluconazole 1 × 800 mg loading, 1 × 400 mg maintenance dose, intravenous vancomycin 4 × 500 mg and intravenous cefoperazone + sulbactam 2 × 2 g treatments were started. We observed *S. apiospermum* in the corneal scraping sample, which the identification was performed by combined phenotypic characteristics and matrix-assisted laser-desorption ionization time-of-flight mass spectrometry on the sixth day of treatment. The drops were revised as fortified vancomycin, ceftazidime, and voriconazole drops 24 times per day. Intravenous voriconazole 2 × 6 mg/kg loading and 2 × 4 mg/kg maintenance dose treatments were started. Three weeks later, left eye visual acuity increased to 20/40, and the corneal abscess regressed. On second-year follow-up, his visual acuity increased to 20/25 for the left eye and the cornea was transparent.

**Conclusion:**

*Scedosporium* group is an opportunistic filamentous fungus that is very rarely seen and causes severe keratitis infections. In the literature, to the best of our knowledge, three cases of keratitis due to *S. apiospermum* after contact lenses were reported, and all were treated with penetrating keratoplasty. In this case, unlike the others, only medical treatment was applied. In cases with suspected fungal keratitis, medical treatment should be started without waiting for the culture result, the findings should be followed and penetrating keratoplasty should be performed in the case of no response to treatment.

## Introduction

Fungal keratitis is an eye infection that is difficult to treat, with a poor prognosis. *Fusarium* and *Aspergillus* are the most common [1]. *Scedosporium apiospermum* is a filamentous fungus formerly known as *Monosporium apiospermum*. *Scedosporium* species are very rare among filamentous fungi causing keratitis. *S. apiospermum* is found on soil, fertilizer, polluted water, spoiled vegetables, and other natural areas. All over the world, it causes mycetoma, which is a chronic subcutaneous infection manifested by the formation of granules that causes severe local or widespread infections in patients with immunosuppression. The most common presentations in the eye are keratitis (84.6%) and sclerokeratitis (15.3%). Risk factors include ocular trauma, immunosuppressive drug use, long-term topical or systemic steroid use, preexisting corneal surface disease, underlying systemic diseases such as diabetes mellitus, and contact lens use [2].

In this study, we reported a case of keratitis due to *Scedosporium apiospermum*, which is seen rarely, after inappropriate contact lens wearing and treated only with medical treatment.

## Case description

**Fig. 1:**
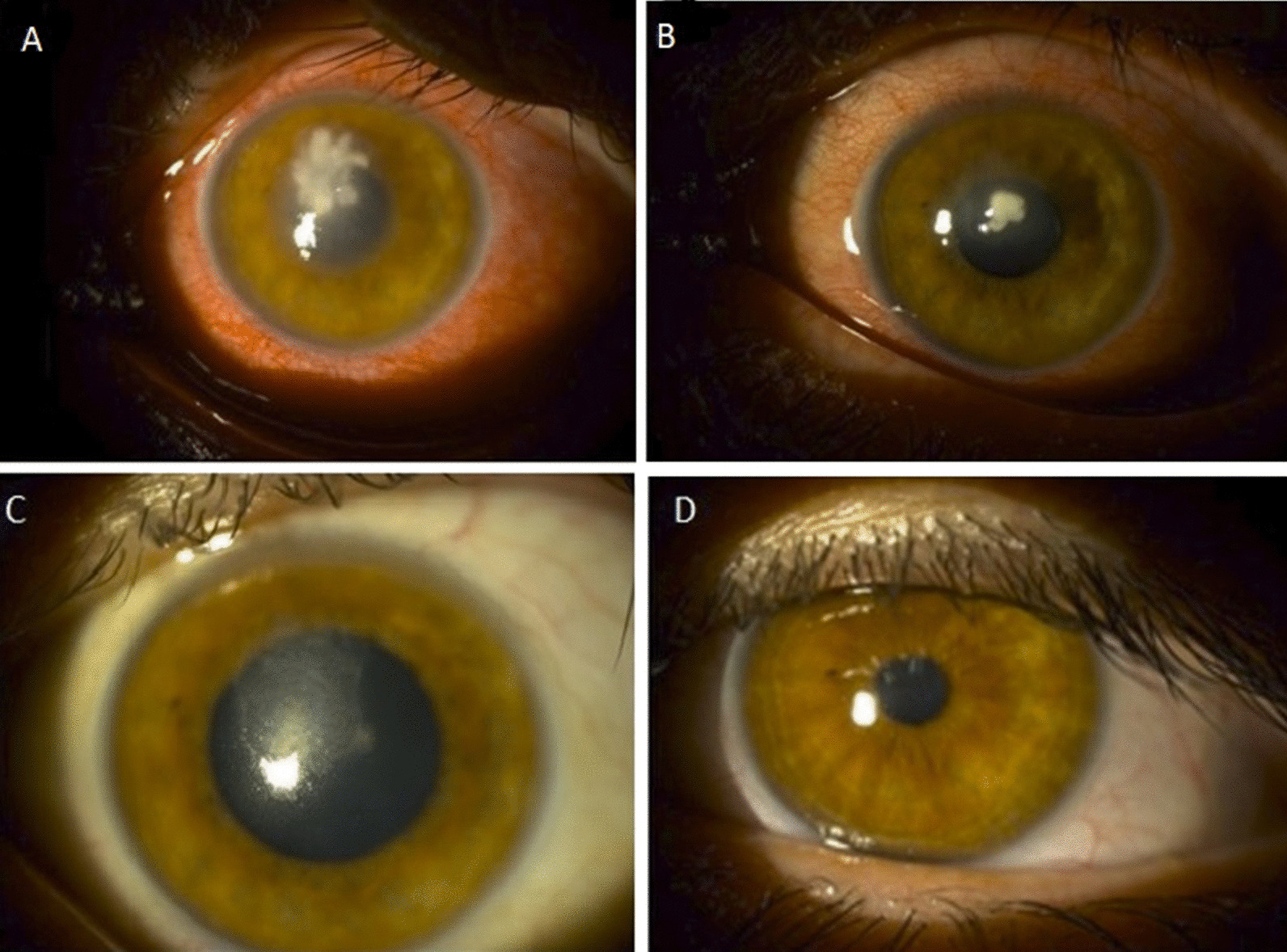
**A** The second day of patient’s treatment, 3 × 3 mm corneal abscess with conjunctival injection, hypopyon regressed. **B** The 22nd day of patient’s treatment, corneal abscess regressed. **C** Two months after treatment, the patient had a minimal corneal haze. **D** Two years after treatment, the patient had a clear cornea

A 34-year-old Caucasian male patient was admitted to our clinic with complaints of pain in the left eye and blurred vision. He had no history of systemic disease, drug usage, or trauma. The patient had a history of wearing contact lenses for 3 years and continuously wearing them for 10–15 days. At the first examination, the patient’s visual acuity (VA) was 20/20 according to the Snellen chart and counting fingers at 30 cm, for the right and the left eyes, respectively, and intraocular pressure was 12 mmHg and 14 mmHg, respectively. On slit-lamp examination, the right eye was normal. There was a 3 × 3 mm corneal abscess at the center of the cornea with a 2 mm hypopyon on the left eye. Fundus examination was normal. Samples of conjunctival swab and corneal scrapings were taken on the same day of the patient’s admission to the hospital. Corneal scraping samples were plated onto Columbia agar with 5% sheep blood (Becton Dickinson, Germany) and Sabouraud dextrose agar (Becton Dickinson, Germany) and incubated at 30 °C under aerobic conditions. Two smears were made for a 10% KOH wet mount and Gram staining. Microscopic examination of the KOH wet mount and Gram staining showed fungal filaments. Empirically topical fortified (FF) cefazolin drops (Cefozin, Bilim İlaç) 24 times/day, FF Gentamycin Sulfate drops (gentamicin 0.3%, DEVA) 16 mg/ml 24 times per day, and fluconazole drops (Fluzamed/World Medicine) 2 mg/ml 24 times per day were started. Intravenous fluconazole (Candisept/VEM) 1 × 800 mg loading, 1 × 400 mg maintenance dose, intravenous vancomycin (Edicin/Sandoz) 4 × 500 mg, and intravenous cefoperazone + sulbactam (1107.4 mg + 1107.4 mg) (Sulzon/Tüm Ekip) 2 × 2 g treatment was started according to infectious disease consultation. The hypopyon regressed on the second day of treatment (Fig. 1A)

**Fig. 2:**
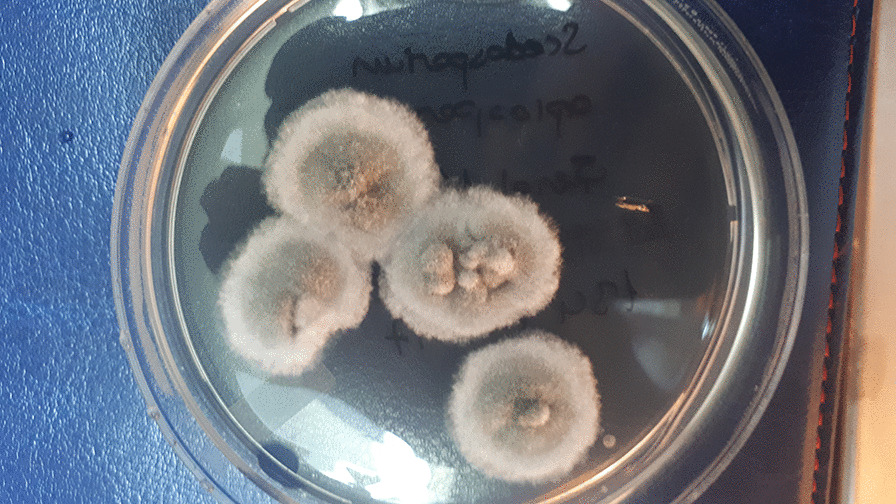
*Scedosporium apiospermum*. Sabouraud dextrose agar, 30 °C, 4 days. Surface of colony

**Fig. 3:**
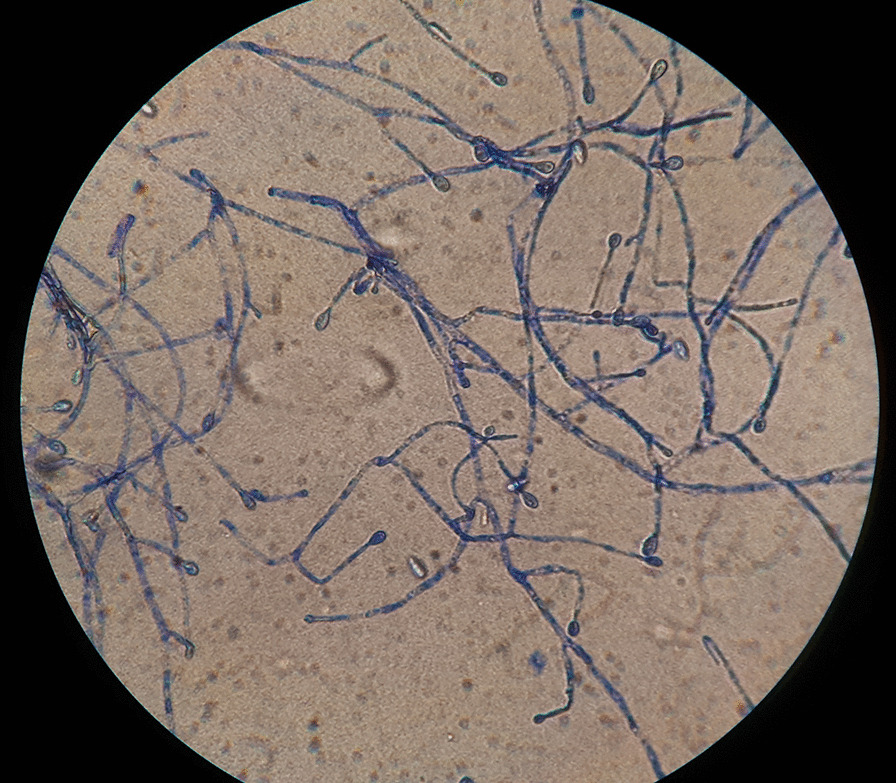
*Scedosporium apiospermum.* Lactophenol cotton blue (original magnification, ×400)

When growth appeared (on the sixth day of treatment), it was examined both macroscopically and microscopically. Species identification was performed by combined phenotypic characteristics and matrix-assisted laser-desorption ionization time-of-flight mass spectrometry (MALDI-TOF–MS) (Bruker Daltonics, Germany). *Scedosporium apiospermum* colony was initially light-colored and later turned gray-brown (Fig. 2). The conidia were ovoid, unicellular, and subhyaline (Fig. 3). Antifungal susceptibility testing was performed by gradient diffusion test (E test, Biomerieux, Turkey) for amphotericin B, the azoles (fluconazole, itraconazole, posaconazole, and voriconazole), and the echinocandins (anidulafungin, caspofungin, micafungin) according to the manufacturer’s instructions. The minimum inhibitory concentrations for amphotericin B, itraconazole, posaconazole, voriconazole, anidulafungin, caspofungin, and micafungin were found to be 0.064, 0.25, 0.032, 0.064, 0.75, 0.125, and 0.032 μg/ml, respectively.

After culture results were obtained, the drops were revised as FF vancomycin (Edicin/ Sandoz İlaç) drops 24 times per day, FF ceftazidime drops (Zidim/Tüm Ekip) 24 times per day, and FF voriconazole drops (Vfend/Pfizer) 24 times per day. Intravenous fluconazole (Candisept/VEM) treatment was stopped, and intravenous voriconazole (Vfend/Pfizer) 2 × 6 mg/kg loading dose and 2 × 4 mg/kg maintenance dose treatments were started. On the 22nd day of hospitalization, the patient’s VA was 20/20 and 20/40 in the right and left eyes, respectively. On slit-lamp examination, we observed a 2 × 2 mm abscess in the left eye (Fig. 1B). After the patient was discharged from the hospital, oral voriconazole (Vfend/Pfizer) 2 mg/ml, 200 mg 2 × 1 treatment continued. Two months later, the corneal abscess regressed with minimal haze (Fig. 1C). On second-year follow-up, the patient’s visual acuity was 20/20 for the right and 20/25 for the left eye, and the cornea was transparent (Fig. 1D) (Table 1).

**Table 1:** Timeline

Time (days)	Day 0	Day 6	Day 22	Day 60	Day 730
Visual acuity (Snellen chart)	Counting fingers at 30 cm	20/400	20/40	20/25	20/25
Findings	A 3 × 3 mm corneal abscess with hypopyon	The hypopyon regressed	A 2 × 2 mm corneal abscess	The corneal abscess regressed with minimal haze	The cornea was transparent
Treatment	FF gentamicin and FF cefazolin drops 24 times per day Intravenous fluconazole 1 × 800 mg loading, 1 × 400 mg maintenance dose Intravenous vancomycin 4 × 500 mg Intravenous cefoperazone + sulbactam 2 × 2 g	FF vancomycin, ceftazidime voriconazole drops 24 times per day Intravenous voriconazole 2 × 6 mg/kg loading and 2 × 4 mg/kg maintenance dose	FF vancomycin, ceftazidime voriconazole drops 8 times per day Oral voriconazole 200 mg 2 × 1	Treatment stopped	No treatment

*FF* fortified

## Discussion

Fungal keratitis is one of the most important causes of ophthalmic mycosis. It is usually characterized by corneal epithelial defect and stromal infiltration. Corneal infection due to *S. apiospermum* was first described in 1955 [3]. While keratitis was detected in the majority of these patients, endophthalmitis, scleritis, corneal ulceration, conjunctival mycetoma, keratouveitis, retinitis, chorioretinitis, and orbital infection were also diagnosed [4].The use of steroids and the presence of immunosuppression from diseases such as diabetes may worsen the prognosis of fungal keratitis [5].

In previous case reports and literature review, a few cases of ocular infection due to *Scedosporium* were observed even in immunocompetent patients, resulting in vision loss or evisceration/enucleation despite medical and surgical treatment [6]. It is also suggested that early diagnosis, along with proper treatment, is the most important determinant of the prognosis. Soft contact lenses are one of the most important risk factors for fungal keratitis in temperate climates [7]. In this case, the main risk factor is improper use of the lens without removing the lens for a long time. Contamination of the cleaning solution may also have caused this condition. As of now, only three cases of keratitis due to *S. apiospermum* have been reported after wearing contact lenses [6, 8, 9]. The first case was treated with topical voriconazole and intravenous voriconazole [6], the second case (with endophthalmitis and had pars plana vitrectomy) was treated with intravenous voriconazole and intravitreal voriconazole [8], and the last case was treated with topical econazole (1%) and amphotericin B (0.15%) with oral itraconazole (200 mg daily) [9]. All three cases underwent penetrating keratoplasty. In this case, unlike other cases, keratitis regressed only with medical treatment.

In a study by Ramakrishnan *et al*. [10], *Scedosporium* was detected in 13 (0.8%) of 1621 patients with culture-positive fungal keratitis. The combination of natamycin and fluconazole was successful in seven out of ten patients. In total, 30.7% of these patients have trauma due to a nonspecific foreign body, 15.3% trauma with organic material, 7.6% have uncontrolled diabetes, and 7.6% have a recent history of cataract surgery. In five cases, no cause was found (38.46%). Nulens *et al*. [11] showed that, after 12 days of oral voriconazole treatment, the level of voriconazole in the aqueous humor reached 53% of the plasma. In that case, it was reported that, as there was no response from itraconazole and amphotericin B treatment, the patient was treated with penetrating keratoplasty and oral voriconazole treatment was initiated. In another study [12], 1% topical voriconazole monotherapy was found to be effective in *Scedosporium* keratitis accompanied by mild anterior chamber inflammation. Özkan *et al*. [13] report a case that was successfully treated with 1% voriconazole eye drops from the third day of prediagnosis.

## Conclusion

We reported the case of a patient who developed fungal keratitis after wearing contact lenses, which is rarely seen. After *S. apiospermum* was detected in culture, the treatment of the patient, who responds to topical and intravenous voriconazole treatment, was continued. In patients with fungal keratitis or endophthalmitis, medical treatment should be started urgently, the findings should be followed daily, and if there is no response to treatment, keratoplasty should be performed without delay. The patient’s findings improved with medical treatment. In addition, fungal factors should be taken into consideration in cases followed up for keratitis (especially for contact lenses), and microbiological examinations should be performed accordingly.

## Data Availability

Figures were added as supporting data.
